# Are Visual Peripheries Forever Young?

**DOI:** 10.1155/2015/307929

**Published:** 2015-04-06

**Authors:** Kalina Burnat

**Affiliations:** Laboratory of Neuroplasticity, Department of Molecular and Cellular Neurobiology, Nencki Institute of Experimental Biology, Pasteur 3, 02-093 Warsaw, Poland

## Abstract

The paper presents a concept of lifelong plasticity of peripheral vision. Central vision processing is accepted as critical and irreplaceable for normal perception in humans. While peripheral processing chiefly carries information about motion stimuli features and redirects foveal attention to new objects, it can also take over functions typical for central vision. Here I review the data showing the plasticity of peripheral vision found in functional, developmental, and comparative studies. Even though it is well established that afferent projections from central and peripheral retinal regions are not established simultaneously during early postnatal life, central vision is commonly used as a general model of development of the visual system. Based on clinical studies and visually deprived animal models, I describe how central and peripheral visual field representations separately rely on early visual experience. Peripheral visual processing (motion) is more affected by binocular visual deprivation than central visual processing (spatial resolution). In addition, our own experimental findings show the possible recruitment of coarse peripheral vision for fine spatial analysis. Accordingly, I hypothesize that the balance between central and peripheral visual processing, established in the course of development, is susceptible to plastic adaptations during the entire life span, with peripheral vision capable of taking over central processing.

## 1. Introduction

For decades most of the visual research has been focused on high acuity central vision, and as a result the role of peripheral vision is underestimated (e.g., [1–4]). For instance, we only recently learned that information about the position of recognized objects within visual space is important and stored in working memory (reviewed in [5, 6]). By this review I would like to highlight the plasticity potential of visual peripheries. Most of the visual plasticity models are based on results solely deriving from the central visual field, whereas peripheral vision not only covers a large part of the visual field but also actively participates in attentional selection of visual space to be processed by central vision. Ontogenetic and phylogenetic descriptions of the visual system made me hypothesize that the peripheral retina and the entire peripheral visual system have immature features. According to the concept proposed here, the immaturity of peripheral visual system would be a favorable condition for maintenance of high level of plasticity throughout life.

**Figure 1:**
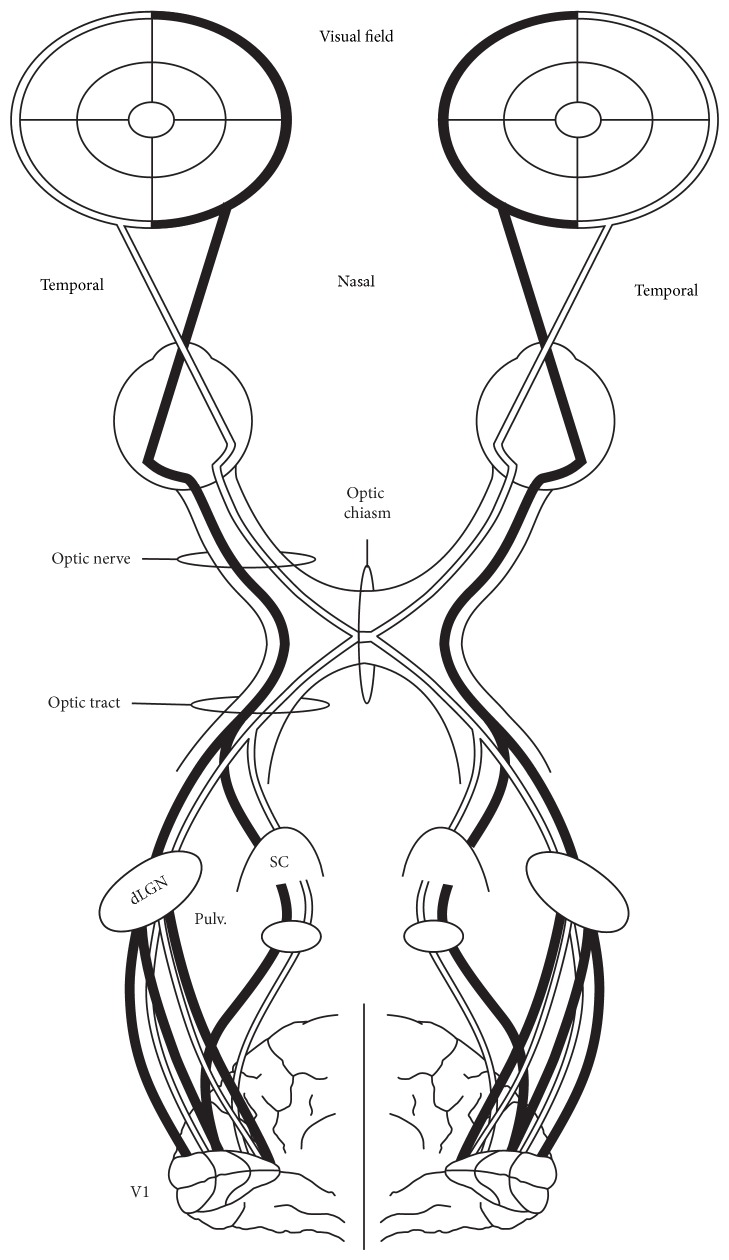
The main projection pathways of the primate visual system. Temporal retina receives visual input from the nasal half of the visual field, whereas nasal retina receives input from the temporal half of the visual field. The optic nerves deriving from the temporal half of the retina (black line) project ipsilaterally, whereas the nasal nerves (white line) cross at the optical chiasm and project to the contralateral hemisphere. Most of the visual fibers reach the visual cortex through relay synapses located at the dorsal lateral geniculate nucleus (dLGN) in the thalamus. A smaller percentage of visual fibers reach the primary visual cortex (V1) through the superior colliculus (SC) and pulvinar (Pulv.).

**Figure 2:**
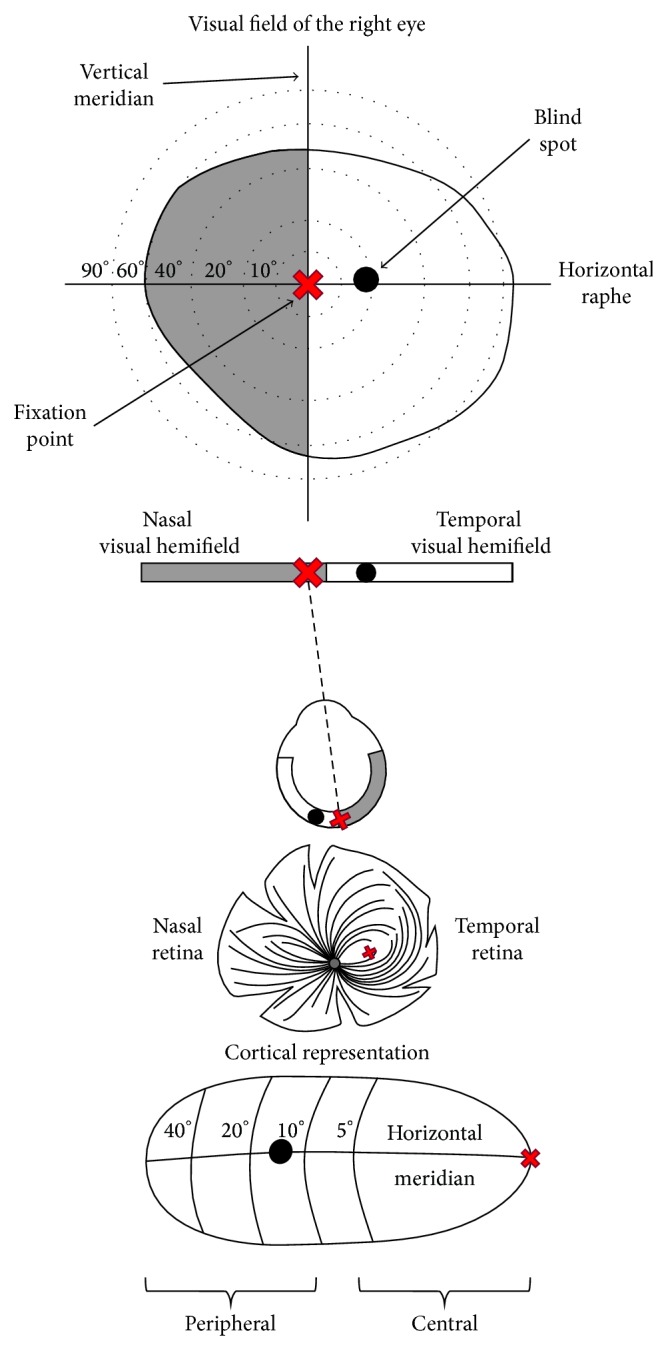
Projection representation of the right visual field. Right eye receives input from nasal visual hemifield (grey) with representation of the central fixation point (red cross) and temporal visual hemifield (white) with blind spot (black dot) located at visual 10°. The thick irregular black line delineates part of the visual field as seen through the right eye. Retinal representation of the visual field shows position of the central fixation and blind spot. Temporal retina receives input from the nasal visual hemifield (gray) and nasal retina (white). Drawing of the flatmount retina preparation shows optical nerves encircling* area centralis* with central fixation point (red cross), with all optical fibers and blood vessels leaving the retina via blind spot. In the cortical representation of the visual field, note the magnification of the representation of the central visual field (compromising visual 5°) and the relatively small peripheral visual field representation.

**Figure 3:**
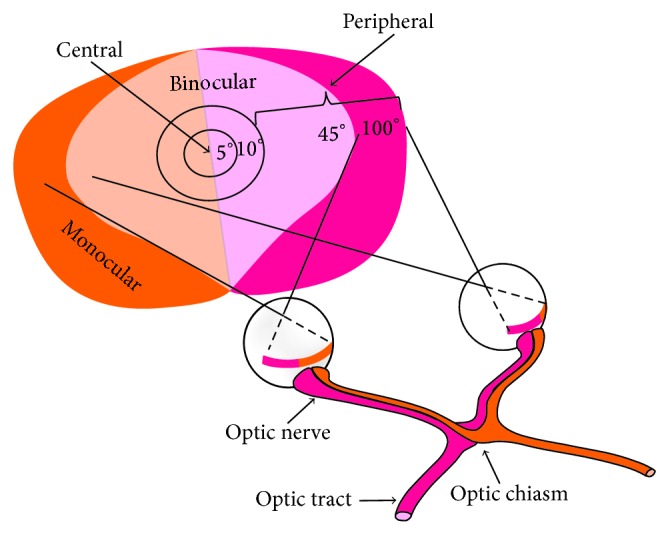
Scheme of the visual field when tested binocularly. Right hemifield is marked in pink and left in orange. Binocular visual field is represented in lighter shade and monocular in darker. Visual input from each half of the visual field after crossing of optic nerves at the optic chiasm is projected via optic tract to the contralateral hemisphere.

Extracting information regarding the peripheral visual system from the literature is not always straightforward, especially since published investigations are not typically focused on comparisons between the peripheral and central visual system. Instead, they either concentrate on separate ganglion cell classes or use retinal regional divisions: temporal regions comprising fovea and nasal retinal regions and their projection zones (see Figure 1 for a comparison of nasal and temporal visual field projection zones in primates). Comparing anatomical and electrophysiological results with psychophysical data is even more confusing, as investigations of the temporal visual field give information about the quality of projections from nasal retina and investigations of the nasal visual field describe temporal projections (see Figures 2 and 3 for a comparison of visual field depictions with depictions of retina and cortical representation). I consider (if not mentioned otherwise) central processing as the cortical representation of fovea including 5 visual degrees and beyond 10 degrees as peripheral processing.

Within the visual cortex, as a general rule, there is a gradient between central and peripheral visual processing, with a sharply defined representation of the central, foveal high spatial resolution occupying only 5 degrees of the central visual field, having spatial thresholds even smaller than a cone diameter [7], and peripheries with poor spatial resolution but high sensitivity for motion (Figure 2 and [8, 9]). Adult-like central-to-peripheral gradient of retinal ganglion cell density, cell body, and dendritic field size is mirrored by the central-to-peripheral gradient of its representation at the subcortical and cortical level. The primary visual cortex exhibits the well-described disproportionate overrepresentation of the central region of the retina, as compared to the underrepresented far periphery due to the number of afferents from the respective retinal regions (Figure 2). The cortical magnification concept substantiates how visual information from one degree of the visual field projects to the primary visual cortex (reviewed in [10]). Similarly, higher visual areas are arranged accordingly with central-to-peripheral bias, where regions discriminating objects with high acuity load such as faces are stimulated by central locations and regions involved in discrimination of crude, large objects such as buildings are stimulated by peripheral locations [11].

## 2. The Functional Significance of Peripheral Visual Processing

During normal daily life the relative position of central and peripheral visual fields within an observed visual scene is constantly changing, as our eyes are hardly ever at one stable position. Instead, eyes constantly explore the visual scene with saccade movements. Visual spatial attention selects fragments of the visual scene (percepts) for further detailed analysis and then directs saccades towards objects to be examined in detail by central vision. The selection process depends on the attentional load of each percept, defined by different physical properties or even memory trace, as substantiated by the concept of salience mapping [12]. Though the neural circuits accounting for visual attention are complex and not yet fully established (recently reviewed in [13, 14]) it is well recognized that shifts of attention are coordinated by peripheral vision [15].

Peripheral and foveal analyses occur in parallel, with reciprocal influence depending on the attentional load of each percept location [16]. Attention can influence visual processing throughout visual areas to a different extent, as shown with measurements of fMRI responses collected during a luminance detection task with checkerboard patterns. Namely, centrally located cues induced attentional enhancement within the primary visual area and higher ventral stream areas, whereas peripherally located attentional enhancement had a beneficial effect within motion-sensitive dorsal stream areas [17]. In the macaque primary visual cortex, directing attention with a cue towards stimuli locations made bar length neuronal tuning more precise at peripheral locations, but not at foveal locations [18]. However, recent work by Ludwig and colleagues [19] indicates that foveal analysis and peripheral selection operates independently, as changing the perceptual difficulty of foveal discrimination of grating orientation did not influence peripheral discrimination.

The position of a stimulus within the visual field determines its attentional load, while neuronal responsiveness exhibits central-to-peripheral gradient across eccentricities of the visual field depending on the physical properties of the tested stimuli. Peripheral percepts are strengthened with increasing stimulus size and/or velocity, which optimizes them for attentional redirection of foveal analysis to suddenly appearing objects. Specific for peripheral processing, motion analysis also shows a central-to-peripheral gradient, with velocity sensitivity that shifts from slow to faster velocities with increasing eccentricity [20] and relative motion detection that is characteristic for central visual field locations [21]. This specialization is not surprising, as the peripheral retina is dominated by motion-sensitive Y-type neurons [22, 23] that project to the peripheral visual field representation in the primary visual cortex. In contrast to the abundance of information about the neuronal properties of the mammalian peripheral retina, less information is available on peripheral cortical processing. In adult marmoset, V1 neurons representing the peripheral visual field, similar to their retinal counterparts, were also shown to be more specialized for motion processing than neurons in the central visual field representation that process high acuity vision [24, 25]. The dominating population of neurons located within the peripheral visual field representation in cat area 17 is also motion sensitive [26–28].

Although peripheral visual processing is coarse and does not imply high spatial resolution, perception of faces is the exception, as they are identified even at the visual peripheries. There is emerging evidence that perception of faces shows a peripheral detection advantage, but only when faces are presented in a brief flash or between flanks ([29]; presentation at 16 visual degrees). Notably, humans detect fearful emotional facial expressions even if presented at the far periphery, up to 40 degrees of eccentricity [30, 31]. Prostriata, an evolutionarily ancient limbic area, were recently visually characterized in marmoset as a potential link between the visual and limbic system that operates as fast recognition of emotional signals. The prostriata are located between the primary visual cortex and the hippocampal formation and have solely periphery-driven visual responses [32]. Due to interconnections with various sensory and association cortical areas, prostriata have thus far been regarded as a part of the retrosplenial cortex (reviewed in [33]). Recent data shows that prostriata neurons have latencies similar to V1 neurons and that their visual responsiveness is limited to stimuli located solely within the peripheral visual field, suggesting a separate function in monitoring peripheral visual space for novel stimuli [32, 33].

## 3. Development of Peripheral Processing Takes More Time

During the development of the visual system, the quality of vision has a key role in structuring neuronal circuitry. Importantly, the development of motion and fine detail sensitivity are separated in time (reviewed in [34]). At birth newborns have blurred vision and achieve the emmetropic state during development, allowing eyes in a relaxed state to see objects at far distances in sharp focus, whereas sharp vision of close objects requires accommodation. Paradoxically, peripheral visual inputs control the establishment of foveal sharp vision which depends on the developmental process of emmetropization [35].

**Figure 4:**
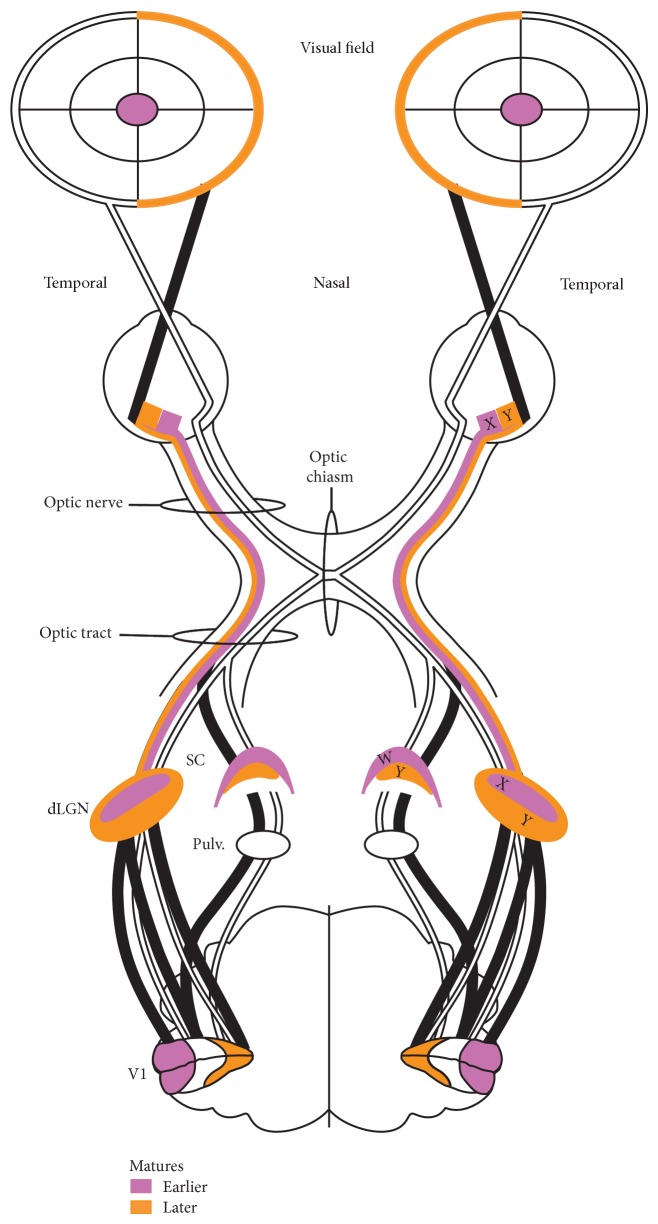
The main projection pathways of the carnivore shown as a compilation of developmental data. Parts of the scheme that are marked in pink develop earlier than those marked in orange. First visually evoked responses derive from central visual field (cats: [57]; humans: [58]); peripheral locations within nasal visual hemifield evoke responses later (humans: [57]; cats: [96]). Central-to-peripheral developmental gradient is shown in the retina (multispecies review [49]) and at the cortical level (marmosets: [54]; cats: [55]). X-type cells develop earlier than Y-type in the retina [123] and at the dorsal lateral geniculate nucleus (dLGN) level (humans: [124]; cats: rev. [125]). W-type cells develop earlier than Y-type at the superior colliculi (SC) level [126]. Superior colliculus develops earlier than dorsal lateral geniculate [127, 128]. Ipsilateral projections from peripheral retina develop later (cats: [67]).

In primates ocular growth and refractive development are controlled by peripheral vision, since foveal ablation in normal infant rhesus monkeys does not result in refractive impairments, whereas peripheral defocus with unrestricted central vision is not sufficient to guarantee normal emmetropization [36–38]. Moreover, children with diseases affecting peripheral retina have a significantly higher frequency of refractive errors than children with central vision impairments [39]. Similarly, cats raised in defocus covering the entire visual field did not show any signs of refractive impairments [40], while cats raised with goggles limiting only the peripheral visual field exhibited myopia [41]. In general, features of peripheral vision develop later as compared to those specific for central vision and are more sensitive to developmental impairments ([42], but see also [43]; for anatomical correlations, Figure 4). In cats, velocity and low contrast-defined motion discrimination is impaired when binocular pattern deprivation is induced after the initial two months of normal vision at 3-4 months of life. In contrast, binocular pattern deprivation during the first 2 months of life did not weaken motion perception, revealing the occurrence of a critical period for some aspects of motion perception later in development than was previously suggested [42]. Depending on the velocity of dots tested with coherent motion displays, the directional selectivity of cortical neurons develops early in life [44–46]. However, high velocity tuning specific for peripheral processing [20] develops relatively late. In children, velocity discrimination between high and low speeds remains immature at the age of 5 years [47], whereas sensitivity to the direction of fast motion remains immature at the threshold level even until 12–14 years of age [48].

In the course of postnatal development the cortical representations of the central and peripheral visual fields are not functionally established at the same time, and their formation depends on concurrent retinal development. The sequence of maturation of the central and peripheral visual inputs in carnivores is summarized in Figure 4 (structures and connections that mature earlier are marked in pink and those that mature later in orange). The ganglion cells of the retina mature according to the central-to-peripheral gradient (for a review covering multiple species see [49]). All ganglion cells in the central retinal region are already present in a newborn kitten and reach adult size by P20, while neurogenesis in the peripheral retina continues up to the 3rd week of life [23, 50–53]. The developmental central-to-peripheral gradient is well characterized for retina and yet far less described at the cortical level (marmoset: [54]; cats: [55]). During the early stages of postnatal cortical development both regions are not yet differentiated from each other. Neurons have large receptive fields that are not sharply tuned for orientation of stimuli, thus resembling adult peripheral properties more than central neuronal properties [56].

In accordance with the above-described central-to-peripheral developmental gradient, kittens tested in a perimetrical apparatus show the first visually triggered responses after 2-3 weeks of postnatal development, and these responses are only evoked by large stimuli presented in the central visual field while peripheral stimuli are ignored [57]. The visual field in young children develops similarly [58]. Expansion of the visual field with age most likely reflects the development of attentional processes including the orienting reflex towards peripheries and disengagement of strong attentional load from central fixation stimuli (reviewed in detail by [59]). In cats, the visual field increases at the time when postnatal growth of area 17 takes place (between the 3rd and 6th week of age) and coincides with an increase in the number of new ocular dominance columns [57, 60, 61].

The anatomical and functional formation of ocular dominance columns and the establishment of fine acuity vision have been described in detail for the central visual field representation (V1, area 17; for review see [62]). Ocular dominance column formation begins in the 2nd postnatal week in the central representation (cat: [63]), whereas information about the formation of ocular dominance columns in the peripheral visual field representation is more tentative. Ocular dominance formation in the peripheral region most likely starts later than in the central region since monocular deprivation from eye opening (P8–10) in cats induces ocular dominance plasticity in the central region, while monocular deprivation in the peripheral region only has effect when deprivation starts after the first month of life ([64] compared with [65]). Another indicator of the slower development of peripheral area 17 is the greater developmental synapse elimination in central than in peripheral area 17 between the age of 2 and 7 months [66]. These findings are not surprising when considering the central-to-peripheral development of the retina.

It is obvious that the quality of vision depends on how projections from the retina are formed. As far as I know, there is no data that directly shows a distinction between the developmental timing of nasal and temporal projections in primates. In cats, one finding again puts peripheral cell populations as the ones that develop later in time: temporal ipsilateral connections deriving from peripherally located ganglion cells are generated later than centrally located cells [67]. This result is substantiated by a specific deficiency in orienting toward peripheral locations within the nasal visual field processed by temporal retina in young children [58]. Our recent developmental screening of the cat primary visual cortex using changes in the expression pattern of the activity reporter gene* zif268* did not show an obvious difference in the normal maturation speed of central and peripheral visual field representations. Nevertheless, adult-like features were first detected in the central region whenever there were indications of uneven maturation [55], in line with the swifter maturation of the central part of marmoset monkey primary visual cortex, as visualized by neurofilament immunoreactivity patterns [54].

## 4. Peripheral Vision Maintains a High Level of Plasticity throughout the Lifespan

Features of carnivore peripheral vision shared by the entire retina during early stages of postnatal development are also characteristic for the animals with simpler visual systems, such as fish and even rodents. In contrast, binocularity is one of the key features of the highly specialized adult human and primate visual system, with well-defined foveal central and peripheral retinal inputs. The degree of binocular vision depends on the placement of the eyes and the presence of ipsilateral projections (reviewed in [49]). Less structured rodent vision with laterally placed eyes has a small cortical binocular zone. In mammalian retina, most of the ganglion cells originating from temporal and nasal retina project contralaterally, while in the temporal retina the percentage of ganglion cells projecting ipsilaterally increases from a small percentage in rodents up to the entire ganglion cell population in humans and primates (Figure 1). In rodents retinal visual input shows strong contralateral bias, with visual evoked potential (VEP) amplitudes that are twice as large in response to stimulation of the contralateral eye as to the ipsilateral response (reviewed in [68]). Furthermore, the central peak of cone and rod density in mice is similar to the photoreceptor distribution of peripheral retina in macaque and even cats [69–71]. Importantly, the next similarity between mouse retina and the carnivore peripheral region of the retina is its negligible anatomical differentiation of retinal ganglion cells, where neither soma nor dendritic tree size increases with eccentricity and ganglion cells have relatively large receptive fields [72].

Even in the mouse visual system, the cortical peripheral monocular zone exhibits intrinsic plasticity response to visual manipulations more strongly than the central binocular zone [73]. Such plastic adaptations in the mouse visual cortex are mediated by the robust multisensory response of auditory and somatosensory inputs, which become active after removing visual input during the early stages of development [74, 75] and in adulthood [76]. Multimodal response to the removal of one of the sensory inputs is also well described in humans and in higher animals like cats (reviewed in [77, 78]). In primates and cats, auditory activation of the visual cortex upon binocular deprivation is limited to the cortical peripheral visual field representation (recently reviewed in [77]), in line with auditory afferent input exclusively targeting the peripheral visual field representation (primates: [79]; cats: [80]). Such auditory activation within the peripheral visual cortex is described in normally sighted humans while attending to sound sources outside the visual field [81]. Moreover, it was recently described that auxiliary sounds enhance visual detection solely at the peripheral locations [82]). On the other hand, in deaf subjects the peripheral visual cortex shows stronger sensitivity to visual stimulation than in normal hearing people ([83–86]; reviewed in [78]), leading to retinal adaptations as measured by optical coherence tomography [84].

The multimodal response within the peripheral visual field (as described above) may represent an adaptive mechanism, where the combining of inputs from separate modalities results in the production of a significant signal even if one of the sensory inputs is lost. The peripheral visual system has both old phylogenic and immature features, which may facilitate the upholding of a high level of plasticity throughout the lifespan. As an example of phylogenic old system, the fish retina can be considered as a particular model of everlasting high level of plasticity. Specifically, fish retina has no central vision* per se* and continues to grow throughout the lifespan with retinal ganglion cells added at the peripheral margins throughout the lifespan [87, 88]. In fish, the visual systems ability to adapt to new environments and spatial resolution tuning increases with age, sustaining peripheral-like retinas in an adaptive, plastic stage throughout their lifespan [88, 89]. In contrast, in mammalian retina an adaptive response of retinal ganglion cells to the changing visual environment is documented only during early stages of postnatal development [90–92], while at the cortical level adaptations are well documented in adulthood.

Based on comparative and developmental studies I hypothesize that the visual peripheries are kept in an immature, adaptive state. Results showing developmental improvement of grating acuity and contrast sensitivity in central locations, together with stable levels in the peripheral locations, are interpreted by authors as symptoms of the early maturation of peripheral vision (humans: [93]; macaque: [94]). I have an alternative point of view: if visual processes at the peripheries are relatively constant from birth, then it presumably means that visual peripheries maintain an immature state with a high degree of plasticity throughout the lifespan. Therefore, I propose to interpret such findings as a further confirmation of the general high degree of plasticity of the peripheral visual system, originating most likely as an evolutionary adaptation to risks appearing at the peripheries.

## 5. Early Binocular Pattern Deprivation: Example of Peripheral Vision Deficit?

In their review covering the visual development of deprived children with congenital cataracts, Maurer and Lewis conclude that “visual deprivation interferes with the normal development of the edges of the visual field, with the largest effect on the part of the field that is slowest to develop” [59]. Specifically, the plastic potential of visual peripheries occurring even in late development is reflected by shrinkage of the peripheral visual field in teenagers with obstructed vision due to cataracts [95] and even in cats that are binocularly pattern deprived from birth [96].

Similar to neuronal circuits during highly plastic developmental stages, peripheral vision is vulnerable to changes in the visual environment as shown in clinical studies and animal models of early pattern deprivation. Under normal visual conditions, the peripheral retina of adult cats is dominated by motion-sensitive Y-type neurons that project to the peripheral visual field representations of the dLGN [97]. Long-lasting binocular pattern deprivation (from 5 months up to one year) interferes with this Y-type peripheral domination at the level of the retina [90] and the dLGN [98, 99]. We investigated the influence of binocular pattern deprivation on the development of central and peripheral visual field representation in the primary visual cortex in cats by measuring the expression pattern of genes regulated by neuronal activity. Indeed, our recent observations indicate that 4 months of binocular pattern deprivation from birth appears to hamper the development of the retinal input stream in layers 4 and 6 of the peripheral visual projection zone in cats, but not in the central projection zone in the primary visual cortex [55]. The layers affected by deprivation, that is, layers 4 and 6 in the peripheral primary visual cortex, receive direct thalamic input from Y-type, motion-sensitive dLGN neurons [100–102]. Some of these inputs consist of the uncrossed inputs deriving from peripheral temporal retina, which develop later in time [67]. To make the story complete, the anatomy of retinal ganglion cells deriving from temporal retina, including its peripheral regions, is also affected by long-lasting binocular deprivation [90]. Adult cats deprived from pattern vision during the first six months of life had significantly fewer Y-type temporal retinal ganglion cells at the peripheral locations, and these cells had a significantly larger cell body than retinal ganglion cells in normal cats [90].

The above described functional and anatomical impairments of the peripheral vision upon early binocular pattern deprivation are reflected by the behavioral outcome, that is, specific motion perception impairment [42, 103]. Early long-term binocular pattern deprivation in cat resembles human congenital cataracts which, if left untreated, similarly result in the severe impairment of motion perception [104]. Interestingly, form perception in children with congenital cataracts [105] and in binocularly pattern deprived cats [106] is impaired to a much smaller extent, only at the threshold level.

## 6. Peripheral Vision Can Be Recruited for Fine Vision Analysis

Visual processing trade-offs can be a general mechanism of possible perceptual overrides of central processing by visual peripheries, which can be induced by training even in adult subjects [107]. For instance, it was recently shown that peripheral vision can be recruited for the analysis of a dynamic visual scene in proficient adult basketball players watching video clips of basketball games with selectively obscured central or peripheral vision, but not in less trained players [107].

Directing attention to target locations reduces performance differences between the center and the periphery and improves performance on spatial resolution tasks (for a review see [18, 108]). Attentional shifts from centrally located targets towards peripheries may even successfully increase visual acuity. For instance, [109] describes substantial improvement in an acuity task upon training solely in the peripheries as compared to the foveal location. Unfortunately, the authors considered 5 visual degrees as a peripheral location and 2 degrees as central, and they did not test further locations within the peripheral visual field. This acuity task was based on relative distance discrimination between two squares during foveal fixation, and peripheral improvement could depend on the ability to redirect attention from the fovea to the more peripheral locations or maybe was due to attentional facilitation leading to the loosening of visual crowding. The crowding effect, described as the destructive effect of neighboring objects on discrimination of centrally placed objects, is a characteristic feature of adult peripheral vision and is suggested to be one of the bases for acuity decline with eccentricity ([110, 111], for a review see [10]). Validation of the decrowding related mechanism of acuity task improvement at the peripheral locations was described recently as a long-term adaption to the central retinal scotoma, where in subjects suffering from macular degeneration for many years the peripheral crowding zone resembles that of the normal fovea [112]. Correspondingly, in an artificial viewing situation with obscured central vision, peripheral vision can successfully recognize natural scenes, even if identification depends solely on fine spatial resolution [113, 114].

Artificial central scotoma, or central retinal lesion, is a straightforward experimental procedure that shifts not only perception* per se* but also attention from nonexisting central input to the peripheries. Possible mechanisms of cortical adaptations due to the loss of central vision in animal models and human subjects (reviewed in [115]), along with other implications, include the role of horizontal connections deriving from the intact peripheral visual field representation that surrounds the lesion [116] and age onset [117].

Under normal circumstances the central retina is predominantly associated with acuity processing and the peripheral retina with motion processing. In adult subjects, binocular central retinal lesions induce an instant deactivation of the cortical lesion projection zone, which is partially restored during the months following the lesion [118, 119]. Consequently, damaging central retina leads to dramatic acuity deficits, whereas the outcome for motion has not yet been described [120]. Our preliminary data shows that central binocular retinal lesions in adult control cats resulted in an initial decrease in motion performance followed by a period of significant improvement at 5 weeks after lesion. In contrast, binocularly pattern deprived cats displayed permanently impaired motion performance independent of the central retina damage. Most surprisingly, the spatial frequency thresholds in binocularly pattern deprived cats increased by a factor of 4 in the 3 months after lesion, whereas in control cats the spatial frequency thresholds remained constant. Thus, central retinal lesions in deprived cats may trigger the peripheral retina to recruit the visual system for stationary fine detail analysis [121], especially when taking into account the fact that binocular pattern deprivation is reflected by long-lasting anatomical changes in the neuronal circuitry of the temporal retina, presumably maintaining it at the plastic early developmental stage [90]. The potential for acuity adaptations within the peripheral visual system may be reflected by the relatively large size of the receptive fields of adult peripheral cells. Although this is to my knowledge not directly proven, the peripheral visual receptive fields possibly stay nearly as large as during early stages of development, potentially as a result of the slower development of peripheral retina. Such an idea is particularly appealing since resolution improvement at the peripheries due to the training might be mediated by reduction of size of receptive fields, similarly to well-described neuronal receptive field tuning in the central region of the primary visual cortex that occurs during the critical period (reviewed in [122]).

## 7. Conclusions

Peripheral vision not only covers a large part of the visual field but also actively participates in attentional selection of visual space to be processed by central vision. Ontogenetic and phylogenetic descriptions of the visual system lead me to hypothesize that the peripheral retina and the entire peripheral visual system have immature features. The immaturity of peripheral visual system would be a favorable condition for maintenance of a high level of plasticity throughout the lifespan. I attempted to describe here when and in which conditions peripheral vision has a potential for neuroplastic adaptations. Maybe the balance between central and peripheral visual processing established over the course of development is simply not stable over the total lifespan; can we hope for therapeutic strategies directed at engaging peripheral vision to take over for central vision processing?
